# Assembly Assistance System with Decision Trees and Ensemble Learning

**DOI:** 10.3390/s21113580

**Published:** 2021-05-21

**Authors:** Radu Sorostinean, Arpad Gellert, Bogdan-Constantin Pirvu

**Affiliations:** 1 Computer Science and Electrical Engineering Department, Lucian Blaga University of Sibiu, 550025 Sibiu, Romania; radu.sorostinean@gmail.com; 2 Electronics and Computer Science, University of Southampton, Southampton SO17 1BJ, UK; 3 Industrial Engineering and Management Department, Lucian Blaga University of Sibiu, 550025 Sibiu, Romania; bogdan.pirvu@ulbsibiu.ro

**Keywords:** assembly assistance systems, training stations, decision support systems, decision tree, ensemble learning

## Abstract

This paper presents different prediction methods based on decision tree and ensemble learning to suggest possible next assembly steps. The predictor is designed to be a component of a sensor-based assembly assistance system whose goal is to provide support via adaptive instructions, considering the assembly progress and, in the future, the estimation of user emotions during training. The assembly assistance station supports inexperienced manufacturing workers, but it can be useful in assisting experienced workers, too. The proposed predictors are evaluated on the data collected in experiments involving both trainees and manufacturing workers, as well as on a mixed dataset, and are compared with other existing predictors. The novelty of the paper is the decision tree-based prediction of the assembly states, in contrast with the previous algorithms which are stochastic-based or neural. The results show that ensemble learning with decision tree components is best suited for adaptive assembly support systems.

## 1. Introduction

Efficient production and a fast delivery of products assures a key advantage for companies in the global competition with more demand for mass-customization and shorter product life cycles. Despite automation progress, because of flexibility, complexity and dexterity requirements, manual work remains an important part of production in Europe [1,2] and staff training is an important component of any company’s success. The progress in information technology enabled the development of assistance systems for manual tasks, that will play an important role [3] in an effective training that addresses current challenges such as a high complexity of the products and processes, unskilled employees or a reduced availability of workforce, thus maximizing the potential of manufacturing workers.

For more flexibility of the workers and lower production costs, many factories avoid the full automation, machine-assisted human-centered manufacturing being the common approach. Thus, without full automation, modern factories must adapt to the workers profile for a cost-effective and resource-efficient production. The training stage of workers can be efficient if assembly assistance systems are used [4], because such an assistance allows to better structure, guide and control the manufacturing processes [5], improving the overall performance and reducing the skill requirements of the human workers [6]. The automation should be adaptive to mitigate unbalanced mental workload [7]. The possibility to dynamically configure the level of automation is another necessity [8]. Assembly assistance systems used in monotonous manufacturing tasks should not over-challenge or under-challenge the workers; they must adapt to the constraints of the task [9] and to the needs of the human operator in real-time [10] and must take into account the skills and a possible functional decrease of the human worker capabilities [11].

Thus, interactive and context-aware instructions for the guidance of the workers become more and more important [12]. An important aspect of the assistance systems is to suggest the next best suitable instruction adapted to the worker and to the actual manufacturing state. For this, investigation of prediction algorithms that can be best deployed to model the behavior of workers in manual operations is essential. In the previous work [4], two-level context-based predictors have been studied for such a predictive assembly modeling. In [13,14], Markov predictors were investigated, whereas in [15] the Prediction by Partial Matching (PPM) algorithm was analyzed. In [16], a Long Short-Term Memory (LSTM) was involved to predict the next assembly steps. All these predictors will be further detailed in the next section.

In this paper, to predict the next step in the assembly sequence, an ensemble learning approach is proposed, where the predictions of a group of weak learners are aggregated (i.e., decision trees in our case) to obtain better predictions compared with the best individual predictor [17]. For this, several methods relying on decision trees are discussed in terms of predicting the next step in an assembly process, mainly focusing on classification and regression trees (CART), adaptive boosted decision trees (ABDT), random forest (RF) and gradient boosted decision trees (GBDT). The predictors use as input information the actual assembly state of the target product and some characteristics of the workers. The models are trained to correlate the next assembly state with the above-mentioned input information. For both the training and evaluation stages, the data from two experiments was used: one with inexperienced trainees and the other one with experienced factory workers. In both experiments the target product was a customizable modular tablet, whose components could be assembled in any order. The two datasets were individually used, but also combined in a mixed dataset. The predictor is designed for a sensor-based assembly assistance system. The goal is to find the most performant next assembly step predictor which would be able to model assembly processes, allowing fast adaptation to the workers. The best predictor will be integrated into the final human-oriented assembly assistance system. The novelty of this paper is the assembly state prediction method which in this case is based on decision trees. The previously analyzed predictors were stochastic-based or neural.

The rest of this paper is organized as follows: Section 2. presents the related work, Section 3. describes the proposed methods, Section 4. discusses the results and, finally, Section 5. concludes the paper and suggests possible future work.

## 2. Related Work

### 2.1. Assembly Assistance Systems

Sgarbossa et al. discussed in [18] the importance of assistance systems with individualized capabilities to cover employee skills and needs as part of smart and sustainable anthropocentric systems, while Romero et al. presented in [19] a vision of the future factory worker (i.e., Operator 4.0). Thus, not surprisingly there are several such solutions in the literature, especially having Augmented Reality (AR) as one of the core technologies.

Ojer et al. presented in [20] a projection-based AR solution for assisting operators to assemble correctly printed circuit boards (PCBs), indicating through the conducted usability study that there is significant improvement of operator performance by using the assistance system for new PCBs compared to the traditional system. Pilati et al. described in [21] a real-time assistance system for industrial applications. It has a camera-based markerless motion capture system to track worker movements and an AR application to provide instructions for each consecutive assembly step. The evaluation of the system’s performance indicated a 22% learning rate improvement and a 51% duration reduction of the first assembly process with respect to the classical approach with paper-based instructions. Lai et al. presented in [22] a customizable multi-modal AR-based smart assistance system that includes deep learning features for tool detection enabling on-site instructions and obtained an improvement of approximately 30% in terms of time and errors for assembly tasks compared to the printed manual. Vanneste et al. [23] conducted a study involving 44 operators (i.e., 78% of them with motor or cognitive disabilities) analyzing the effects of oral, paper- and AR-based instructions by setting-up an experiment in which participants had to perform three assembly tasks (i.e., a light fixture, a quality control and support wheel assembly). The authors revealed very good results for AR-based instructions (i.e., a projector) compared to the paper-based approach, especially considering the assembly quality, although AR does not always outperform the traditional method. Even more, compared with audio instructions, AR-based instructions are considered to have less complexity. Moreover, it was observed that the learning curve was improved in case of AR-based instructions, due to lower stress (especially for novices) compared to paper-based and audio instructions. More benefits are also identified in respect to a higher degree of independence and a lower perceived complexity. Finally, the authors revealed that the more assembly trainings are executed, the fewer are the AR advantages. Wang et al. [24] studied what AR-instructions are adequate from a user cognition perspective and proposed a new classification method for assembly processes. After executing a case-study with 23 university students, the authors concluded that the new assembly instructions, which are based on the logical classification of parts, provide better results than conventional assembly instructions, which are classified by means of geometric-level information.

Malik et al. described in [25] a Virtual Reality (VR) based assistance system. It explores an approach for designing human-centered production systems in VR to reduce errors in the design phase of developing human-robot collaboration (HRC) systems. This is done with an initial prototype following a structured framework for using VR in the design of HRC systems that includes a chatbot as virtual assistant. Another VR-based assistance system, designed for the initial training professionals, is presented by Hirt et al. [26]. In their experiment, 21 participants evaluated generally positive the systems on criteria such as familiarity, usability as well as specific VR-metrics (i.e., simulator sickness perception and feeling of presence).

In [27], the authors evaluated a mixed reality guided system in two case studies, one focusing on the interaction technique and the other one on the correct assembly of the motherboard. The mixed reality system scored low among inexperienced users at the usability test while the solution indicated effectiveness compared to the printed manual. How a Cross Reality (XR) assistance system in conjunction with the Industrial Internet of Things (IIoT) can support training of manual operations executed by workers with disabilities together with machines is discussed in [28]. Two user studies involving one manager and ten workers were conducted, to evaluate the information sharing design as well as the system’s usability. Although it is a promising approach, the results revealed the limitations of XR technology applied into the industrial setting, such as the usability difficulties of workers to use devices like Microsoft’s HoloLens.

In [29], Petzoldt et al. compared 15 different assembly assistance systems (research and commercial solutions) and presented the importance of individual adaptation especially in respect of the incentive approach for individual assistance. The general implications and dependencies are discussed regarding a case study which uses a pick-by-light and gamification approach within the assistance system. In [30], Petzoldt et al. detailed a first implementation for assembly assistance, with individual instructions also having a motivational incentive approach.

Although Durlach in [31] indicated “sparse evidence” for assuming that adaptive instruction systems have better outcomes than non-adaptive ones, Gräßler et al. compared the learning effect of adaptive assistance systems (e.g., shortened instruction in the second round) versus non-adaptive assistance (e.g., repetition of the same instructions in the second round) and concluded in [32] that by using adaptive instructions users train faster and do so with fewer errors. The benefit of adaptive instructions is revealed also in [33], where a pilot study in a lab environment was conducted to evaluate an adaptive assistive system that adjusts the assembly work steps according to the worker’s cognitive workload by using four types of biosensors. The authors concluded that a bio-sensor approach for adaptive assistance systems can reduce stress and improve the productivity of operators. Bläsing et al. [34] present the benefits, challenges, and limitations for fusing cognitive ergonomics data with physiological measured data necessary to reduce the cognitive load in assembly systems.

### 2.2. Prediction Techniques

In recent years, decision trees and ensemble learning have become a standard approach to tackle prediction. Problems such as photovoltaic output have been studied in [35] looking for more accurate predictions to the output of solar farms for better grid integration. The problem described by Wang et al. is similar in context to the one presented in this article and further discussed in [14], where the input data is a sequence of events, such as time series in [35] and a sequence of assembly steps in our case. The approach of Wang et al. looks into using a short normalized feature vector in order to predict the energy that is going to be generated by the photovoltaic system in the following days. Given the findings of Wang et al. that GBDT predicts the outcome with the lowest error rate, the model was considered in the present work, too. A similar approach is used by Yang et al. in [36] to predict traffic using GBDT. In [37], Khaleghi et al. used GBDT and the ensemble process to predict the state of health of batteries on electric vehicles and goes in depth into an explanation of the algorithm. In [38], decision trees are used to forecast power line faults.

Decision trees were successfully used for prediction in medicine [39,40,41]. In [42], decision trees were used to predict the pillar stability of underground mines. In [43], Mahmood et al. proposed a decision tree-based model to assist the allocation of new orders in manufacturing networks. In [44], a hybrid predictor based on decision trees was considered for web prefetching. The decision tree uses as features the current link and its type, as well as the predictions provided by Markov models of different orders. The evaluation results have shown the efficiency of the decision tree in comparison with other methods. A review of the decision tree models, with a focus on their performance in prediction, is presented in [45].

Several prediction methods have been applied to provide next assembly step choices within adaptive training stations or assembly assistance systems. The goal is to support the inexperienced workers in their learning stage without a human trainer as well as to assist experienced workers in the manufacturing process. First, a model is built up based on past assembly processes of a certain product and then the model is used to determine the most probable next assembly step, given the current assembly stage. In [4], a two-level context-based predictor is presented, which maintains a left-shift assembly state register in the first level used to select an entry from a pattern history table in the second level containing the next assembly step. In other approaches, a Markov model tracks the occurrence frequencies of multiple possible next states within the pattern history table and, thus, it can provide multiple choices for the next assembly step [13,14]. The PPM algorithm was also evaluated [15] as an assembly step predictor. It combines different order Markov predictors. The PPM of order R first tries to predict with the Markov model of order R. If the Markov model is able to predict, its prediction is returned by the PPM. Otherwise, in the case where the current Markov model cannot predict, the order is decremented until a prediction can be done or all the models were evaluated without success and, in that case, the PPM itself cannot predict. In [16], Precup et al. predicted the next assembly step with an optimally configured LSTM neural network. Among all of the above mentioned assembly modelling techniques, the PPM [15] proved to be the most accurate.

## 3. Next Assembly Step Prediction through Decision Trees and Ensemble Learning

As found in the previous articles [4,13,14], the stochastic approaches (including Markov chains) yield the best accuracy results while the coverage is limited due to the number of scenarios in the training data. The stochastic approaches fail to cover states that were not supplied in the training data, making them less robust when applied to real world usages where new scenarios can be met at any time. To prevent the problem and maximize the prediction rate of the algorithms, a tree-based prediction approach was evaluated.

The algorithms described below were implemented via the help of the popular Python machine learning library Sklearn. Several tree-based prediction algorithms such as CART, ABDT, RF and GBDT were tested to find out which one would best suit the prediction scenario. The novelty of this paper consists in the tree-based assembly state prediction, since all the previously applied methods were stochastic- or neural-based. A comparison with those methods will be presented in Section 4. The goal is to find out the most accurate assembly modeling method to be integrated into the human-oriented assembly assistance system for efficient guidance of the factory workers.

### 3.1. Assembly Assistance System

**Figure 1:**
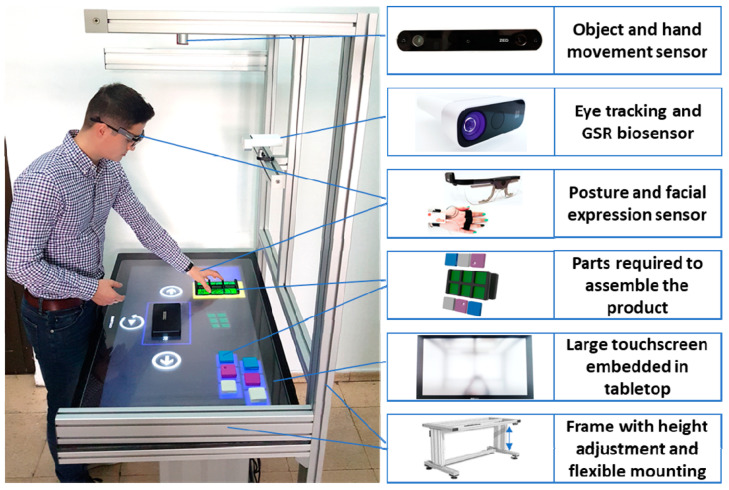
Assembly assistance system.

The assembly assistance system (Figure 1) is a functional prototype designed to show users the correct way to manually assemble products without human guidance. From a conceptual standpoint, it follows a human-centred design approach as defined in the anthropocentric cyber-physical reference model defined in [46]. A key aspect targeted with the assembly system is to expose the trainee to adaptive instructions with respect to type (visual, vocal) and content (detailed, concise), thus customizing the training process to the user’s needs for achieving a good learning result.

The instructions are adapted based on the performance in task execution, operator profile and its current state. For the task performance, data from a 3D stereo camera is used, which enables object and hand movement tracking; moreover, this is complemented by the user interaction with the touchscreen to select the next instruction and thus count the time for the step completion. To detect the operator’s profile (e.g., age, gender, etc.), a front facing camera is used, whereas for the operator’s basic status detection (e.g., angry, happy, sad, neutral, etc.) the images from the front facing camera are used; depending on the experiment and required accuracy, additional data from sensors [47,48] (e.g., microphone embedded in the touchscreen) or biosensors worn by the user (e.g., GSR sensor, eye-tracking glasses, EMG, EEG etc.) can be used to estimate the user’s current state for the assembly instruction’s adaptation. Finally, to enable an ergonomic training, the table has electric actuators for height adjustment. To cover the set-up for different experiments, the mechanical structure enables flexible mounting alternatives throughout the frame.

**Figure 2:**
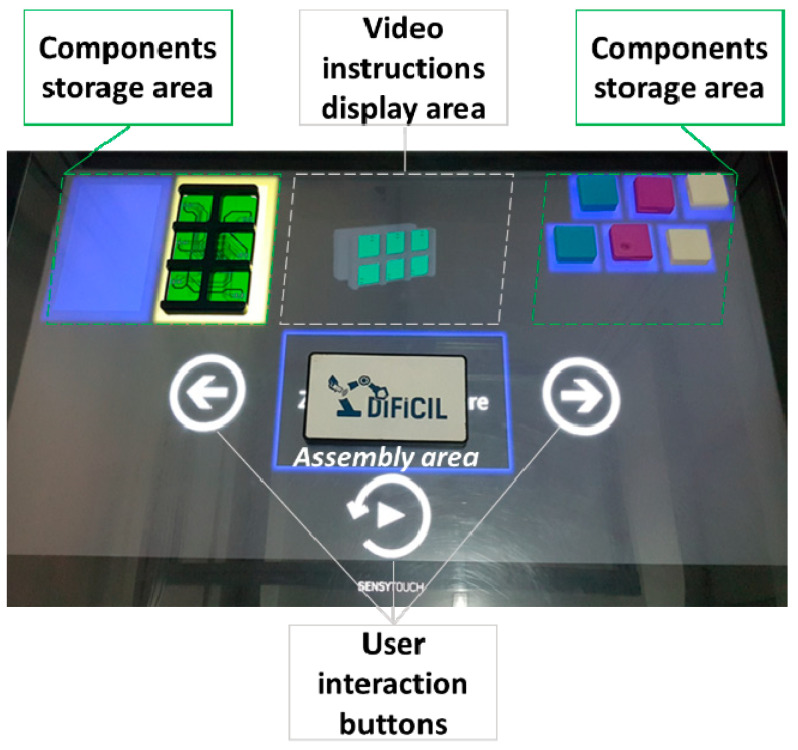
The user interface of the assembly assistance system.

As depicted in Figure 2, all components required for the manual assembly training are situated on the right and left side. The visual instructions are the 3D animations which are displayed in the central part of the large touchscreen. The second visual aid is represented by the areas below each part, which blink when an instruction involving them is active.

Voice instructions explaining the goal of each task are correlated with the visual instructions at each assembly step. The user has three interaction buttons: play / repeat instruction, previous or next instruction. More details about the current assembly assistance system’s implementation and its concept can be found in [15,49], respectively.

### 3.2. Algorithm Design

**Figure 3:**
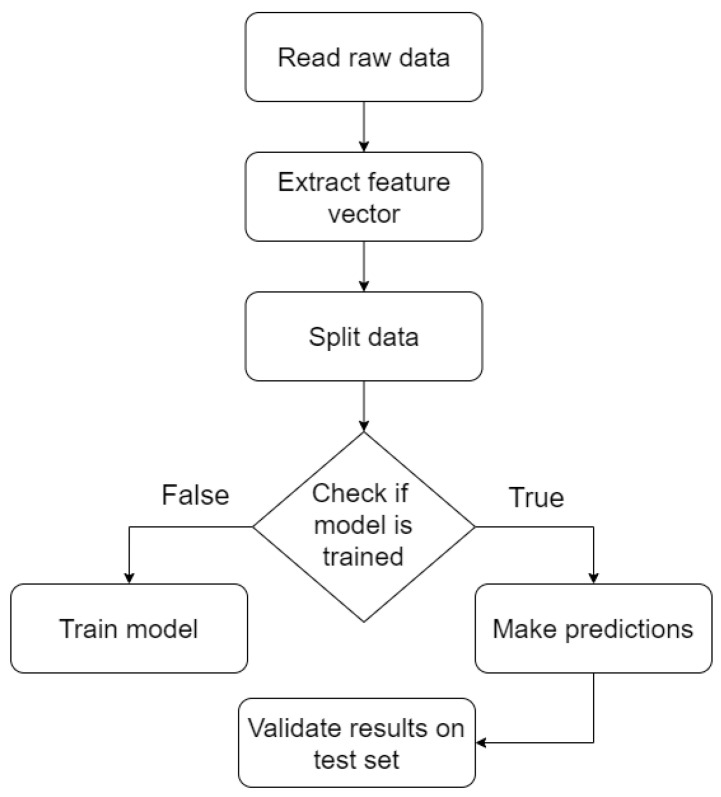
Machine learning pipeline.

The prediction pipeline for the models can be seen in Figure 3, where the data is pre-processed to form the feature vector. After the processing step, the data is split into 75% training data and 25% testing data. While some algorithms like CART take the training data and output a model, RF, ABDT and GBDT have further built in splitting and random sampling that optimize the loss for the training data. The most notable one for this case is GBDT which was used specifically for this feature as it tries to train more decision trees on inputs with high loss where the previous models performed worst, thus making the algorithm an ensemble process.

**Figure 4:**
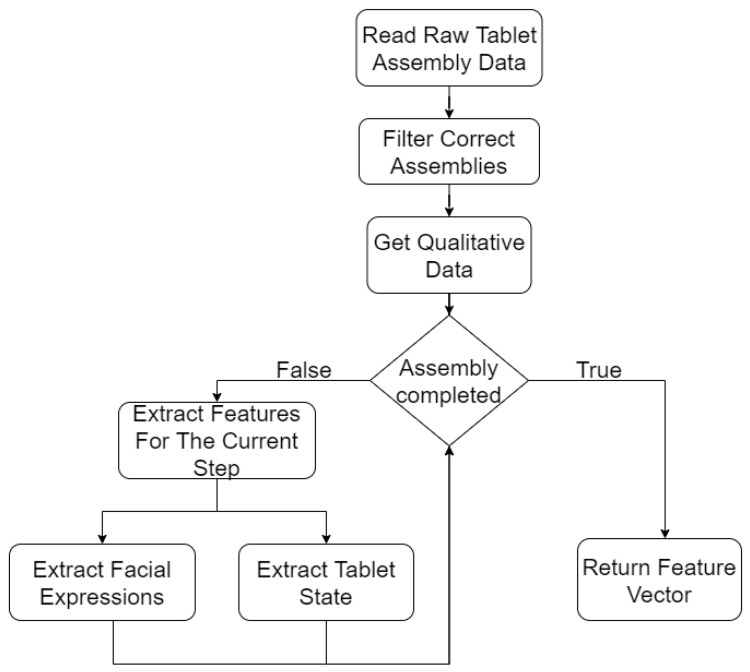
Feature extraction.

The feature extraction is done based on data collected by the different sensors during the assembly process (see Figure 4). The data is filtered, so only correct assemblies remain in order to train the predictors without incorrect assembly sequences. The qualitative data is collected from the users after the assembly process and consist of whether they are wearing glasses, their gender, height and sleep quality. During the assembly, several different sensors track the user to follow each move taken; after each move the facial expression is extracted in order to obtain the emotional status and the tablet state is recorded. Once the final state is reached a feature vector is computed.

After normalizing the individual user data, a feature vector of the form [current_state, height, wears_glasses, gender, sleep_quality] was obtained. The feature vectors were fed into a CART, ABDT, RF and GBDT, in order to obtain the next assembly sequence. All the prediction models use the Gini impurity as criterion:

(1) $$\mathrm{Gini}=1-\sum_{i=1}^{C}{{(p}_{i})}^{2}$$

where c is the number of classes and $p_{i}$ is the probability of an item being chosen; this is done in order to ensure that the class with lowest Gini score is chosen. This will result in features with low Gini score (close to 0) being the most important and defining criteria for the prediction, for example when wears_glasses = 1 the Gini score could be 0.01 and predict that the next move will always be the assembly of the screen module.

#### 3.2.1. Classification and Regression Trees

**Figure 5:**
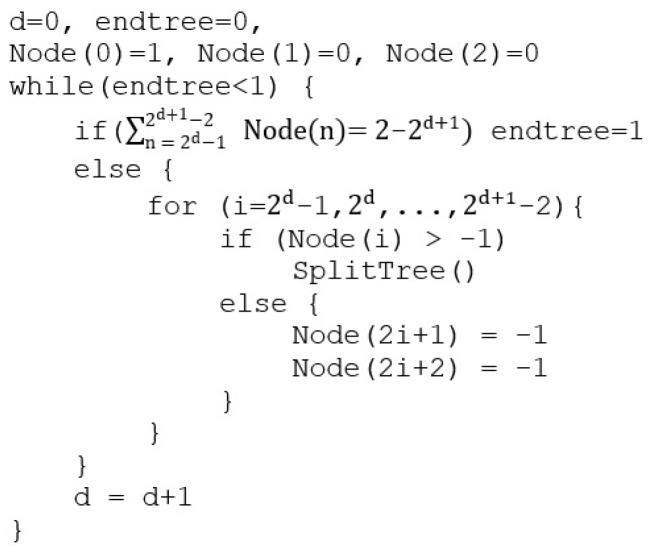
CART pseudocode.

CART is one of the most commonly used approach in machine learning, introduced by Breiman in 1984. CART builds the predictions based on a binary split for each attribute by using regression to find the dependent variable from the set of variables given in the feature vector. The pseudocode for CART is presented in Figure 5, where a feature vector is stored in the root node.

Based on the Gini impurity, the node and its children are split until the leaf nodes reach a Gini score of 0 or cannot be split anymore. CART has a time complexity of $O(f\cdot n^{2}\cdot \log\;n)$, where f is the number of features and n is the number of samples.

#### 3.2.2. Random Forest

**Figure 6:**
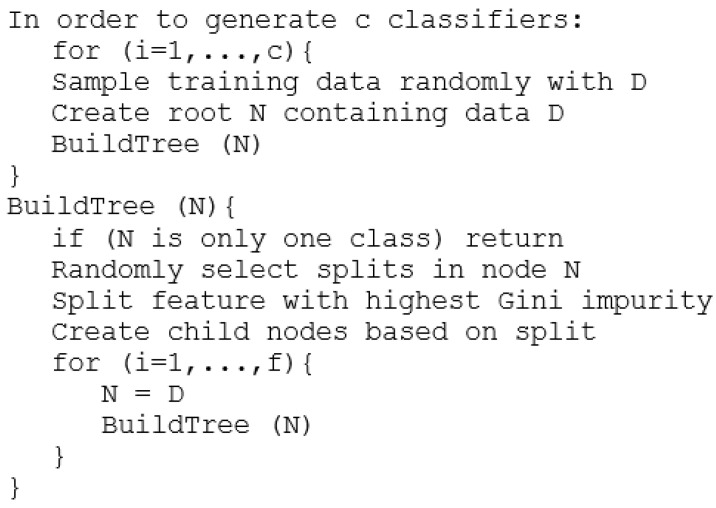
RF pseudocode.

RF is part of the ensemble learning methods; particularly random forest trains many decision trees by taking random samples from the data. This will result in several classifiers, some with better accuracy and results than the others. The result for the prediction or classification done by random forest will use the bagging procedure by averaging the prediction of all the decision trees or by majority voting. The pseudocode for RF can be seen in Figure 6.

RF has a similar time complexity to CART, namely $O(f\cdot n^{2}\cdot \log\;n)$, as RF generates a constant number of trees (given as a hyperparameter).

#### 3.2.3. Gradient Boosting Decision Trees

**Figure 7:**
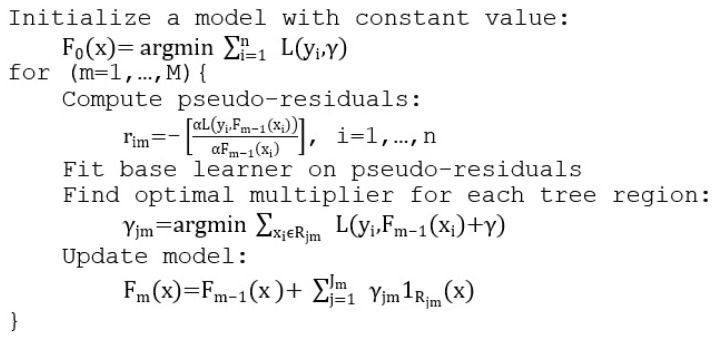
GBDT pseudocode.

Gradient boosting is a supervised ensemble learning method where several weak base learners are trained and the prediction is done via bagging. GBDT focuses on improving each prediction based on the pseudo residuals computed from the previous tree. Thus, the model will fit new decision trees on the data where the previous tree performed badly and the final prediction will be a weighted sum or a majority vote. The pseudocode for GBDT can be seen in Figure 7, where M is the number of iterations, L(y,F(x)) is the loss function, $J_{m}$ is the number of leaves, $R_{\mathrm{jm}}$ is the disjoint region for each leaf at iteration m, $F_{0}$ is the loss reduction function, and α is the learning rate.

The time complexity of GBDT is similar to CART’s and RF’s, namely $O(f\cdot n^{2}\cdot \log\;n)$, as the algorithm generates a set of decision trees (the number of trees being given as a hyperparameter).

#### 3.2.4. Adaptive Boosted Decision Trees

**Figure 8:**
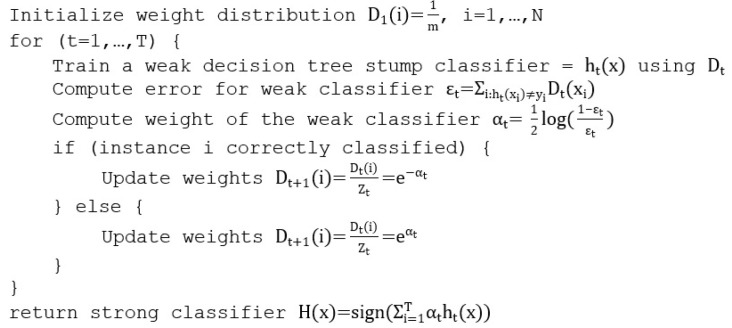
ABDT pseudocode.

ABDT, also known as AdaBoost, is one of the earliest boosting methods used in machine learning. ABDT works on a simple premise: if several weak classifiers that have a prediction power more than random (>50%) are combined, a single strong classifier can be obtained. Each consequent weak classifier is based on a decision tree with a single split, also known as decision tree stump, where ABDT adapts the weight of the stump for the final decision in the ensemble process based on how hard the classification is. The pseudocode for adaptive boosting can be seen in Figure 8, where T is the number of iterations, $z_{t}$ is the normalization factor chosen so that all the weights sum to 1 and $h_{t}\left(x\right)$ is the weak decision tree classifier.

The time complexity of AdaBoost is similar to the complexity of the other tree-based algorithms presented: $O(f\cdot n^{2}\cdot \log\;n)$; although the time complexity is the same, in practice, AdaBoost trains faster as only the stump of the tree is generated instead of the whole decision tree.

## 4. Evaluation

This section presents the experimental methodology and discusses the evaluation of the next assembly step prediction methods proposed in Section 3. The analyzed predictors are then compared with other existing prediction techniques applied in assembly assistance systems.

### 4.1. Experimental Methodology

**Figure 9:**
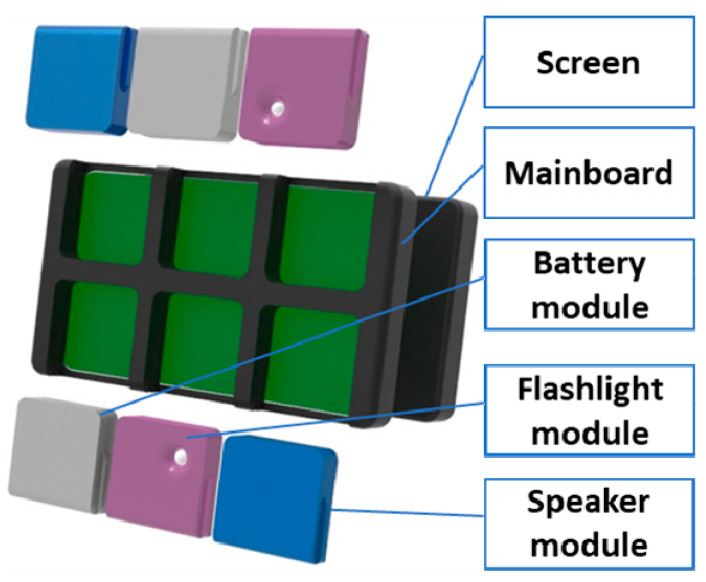
Customizable tablet example.

Two different groups of participants were used to collect the data during the free assembly of a customizable modular tablet (see Figure 9) which has eight components: a mainboard, a screen, two battery modules, two speaker modules and two flashlight modules.

The first group was formed of 68 (2nd year BSc) students inexperienced in such assembly processes and the second group was formed by 111 manufacturing workers with prior experience in such work. The experiment involved a top-mounted camera recording the manufacturing sequences and the assembly station showing on its screen two images of the tablet. The assembly station had marked the initial positions of the components, thus all participants started in identical conditions.

Each participant received an ID. For minimal interaction with the subjects, the experiment was controlled from another room through a laptop connected to the camera. The participants could only listen to a recorded voice message transmitting that “the tablet should be assembled as indicated in the images and that they were required to use all the components in the assembly process”. After the voice message, the participants could start the assembly process. The subjects announced when they finished; the recording was then stopped and they were taken to another room to fill in a questionnaire. The given ID was used to link the answers to the recording. There were questions about human characteristics such as height, age, gender, dominant hand, the highest level of education and if they wear eyeglasses. These questions were followed by others for self-evaluation: “were you hungry during the experiment?”, “do you have any prior experience in product assembly?”, “what was your stress level before the experiment?”, “how would you describe the state you found yourself in during the experiment (at the beginning, during and at the end of the experiment)?”, “are you under the influence of any drugs that might influence your level of concentration?”, and ”how would you describe the sleep quality of the previous night?”

After the experiment, all the assemblies were encoded and each assembly sequence was ranked with a score reflecting the level of completeness. Additionally, for each assembly the duration in seconds was also collected. The dataset collected from the first group with the assembly assistance system [14] is further called “Trainees”, whereas the dataset collected from the second group is called “Workers”. These two datasets were also mixed and randomly sampled in a third dataset for the purpose of the experiment to measure the generalization power of the models. The utilization of such a mixed dataset in the evaluation process is a new evaluation approach in our research. The collected data was processed as in [16] to maintain the same approach and obtain results that can be compared between the algorithms. Following the testing procedures from [16], the results were measured on the dataset that was split into 75% for training and 25% for testing, by using the same metrics: the prediction rate, the coverage and the prediction accuracy. The prediction rate is the percentage of predictions done out of all the assembly steps from the testing set. The coverage is the percentage of correct predictions out of all the assembly steps from the testing set. The accuracy is the percentage of correct predictions out of the predicted assembly steps from the testing set.

### 4.2. Experimental Results

For all the tree-based algorithms, a random grid search was performed to find the optimal parameters for the models. After finding the optimal parameters, a further iteration was done via grid search to obtain the best parameters for the scenario laid out in [14].

Feature importance [50] is calculated based on the Gini importance with the assumption that the tree is balanced:

(2) $$n_{j}=w_{j}C_{j}-w_{\mathrm{left}(j)}C_{\mathrm{left}(j)}-w_{\mathrm{right}(j)}C_{\mathrm{right}(j)}$$

where $n_{j}$ is the importance of node j, $w_{j}$ is the weighted number of samples that reach node j from the feature vector, $C_{j}$ is the impurity value of the node based on the criterion (Gini in our case) and left(j) or right(j) are the child nodes of j to the left and right of the split. To calculate the feature importance for all the nodes we use the following formula:

(3) $$I_{i}=\frac{\underset{j\;\in \;\mathrm{split}\;\mathrm{feature}\;i}{\sum n_{j}}}{\underset{k\;\in \;\mathrm{all}\;\mathrm{nodes}}{\sum n_{k}}}$$

**Figure 10:**
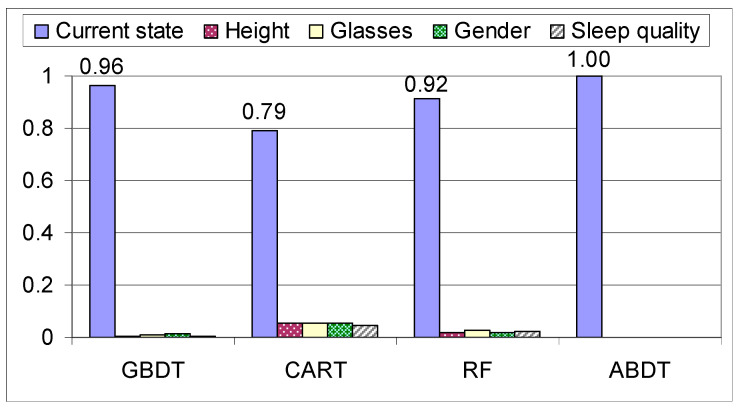
Feature importance on the mixed dataset.

For RF, ABDT and GBDT, where more trees are generated, the feature importance is computed as the sum of the normalizations divided by the number of trees that were generated. In general, the feature importance depicted for the different models in Figure 10, shows that accuracy increases as the score of the additional features decreases. ABDT is the only algorithm that does not follow the general rule, this is due to the fact that during training it generates decision tree stumps instead of an entire decision tree, causing the algorithm to have a higher bias towards the weights of the early states in the assembly phase and ignore the states down the line, which can also be seen in Figure 10. The features are as follows: current state, height (1 for equal or over 174 cm and 0 for under), wears glasses or not, gender (0 for female, 1 for male), sleep quality (0 for sleep quality below 3 and 1 for above or equal). This shows that the main factor for prediction is the current state, the other features having a very low impact on predicting the next move. Thus, as presented in [14], the stochastic methods such as the Markov model, that use the state as base information, have high accuracy.

**Figure 11:**
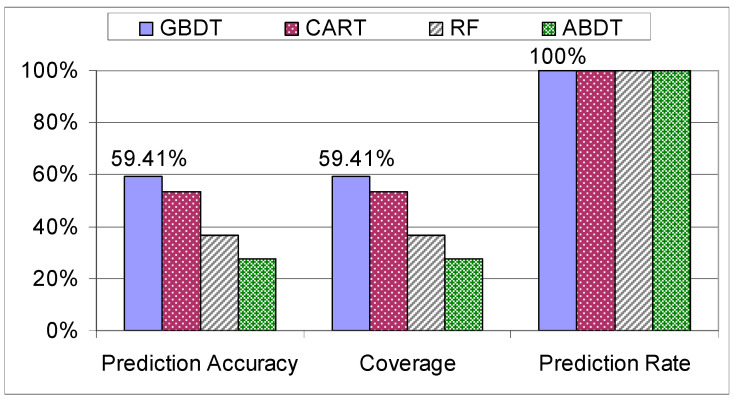
Evaluation of decision trees on the “Trainees” dataset.

The first step that was taken in verifying the results produced by the models was to check how they behave when they are trained and tested on the individual datasets. Unsurprisingly, the results for the “Trainees” dataset have the lowest coverage and accuracy out of all the results (see Figure 11). This is because the “Trainees” dataset is composed of 68 students with no prior experience, thus the assembly process had a higher chance to be different for each student as they were not approaching the assembly in a systematic way, giving the data and the assemblies a higher variance compared to the “Workers” dataset.

**Figure 12:**
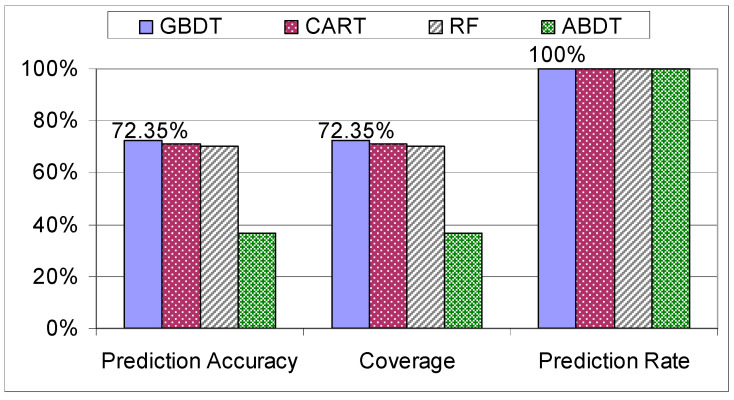
Evaluation of decision trees on the “Workers” dataset.

When the models were examined on the “Workers” dataset, the algorithms started to pick up the systematic way of assembling the tablet, specific to this dataset due to the experience in assemblies, resulting in a peak coverage of 72.35% (see Figure 12).

After examining both scenarios, it was decided to mix the available datasets to use both characteristics of the datasets, namely, high variance from “Trainees” and the systematic assembly from the “Workers” dataset.

**Figure 13:**
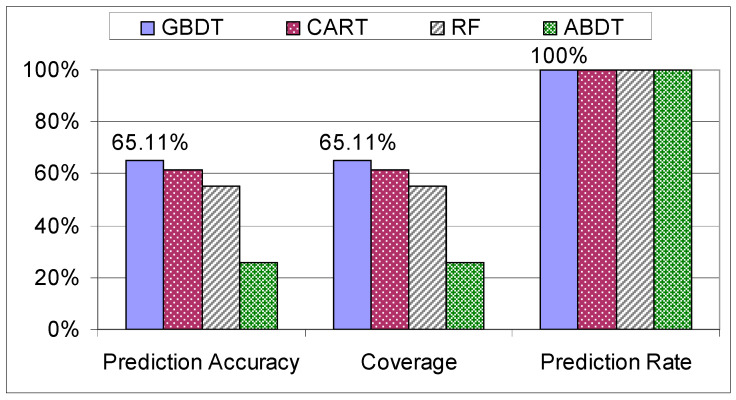
Evaluation of decision trees on the mixed dataset.

As we can see in Figure 13, the model with the highest accuracy out of the ones explored in this paper is GBDT, although unexpectedly CART outperforms RF and this can be attributed to the fact that the data is sparse and RF will produce an invariant tree when the subsample is selected. All the evaluated methods based on decision trees have a prediction rate of 100%, as any input has an outcome. The ABDT model proved to be the weakest tree-based prediction algorithm, its accuracy and coverage being half the accuracy and coverage of the other decision tree models, due to the decision tree stumps used in the process. Next, the GBDT is compared with the Markov model, the PPM algorithm and the LSTM.

**Figure 14:**
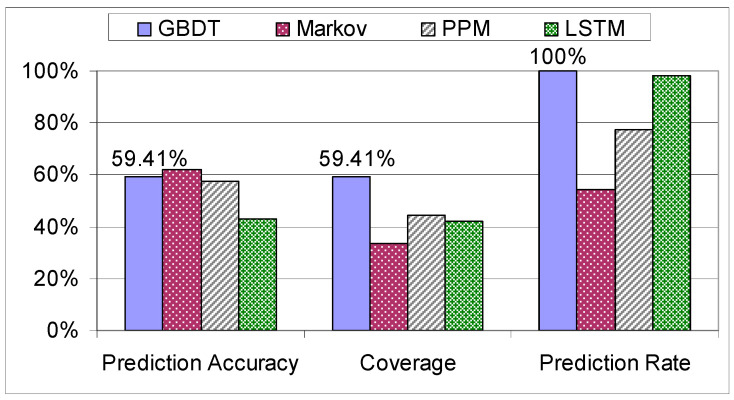
Comparison on the “Trainees” dataset.

**Figure 15:**
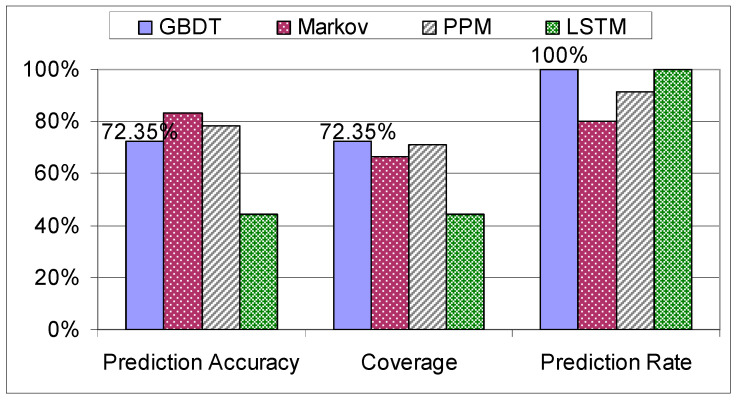
Comparison on the “Workers” dataset.

Analyzing Figure 14 and Figure 15, it is obvious again for all the models that the experienced factory workers are more predictable than the trainees. The differences in the prediction performance of these two categories are high in the case of GBDT and the stochastic models and lower in the case of the LSTM. Thus, the LSTM is not able to exploit the systematic manufacturing style of the experienced factory workers.

**Figure 16:**
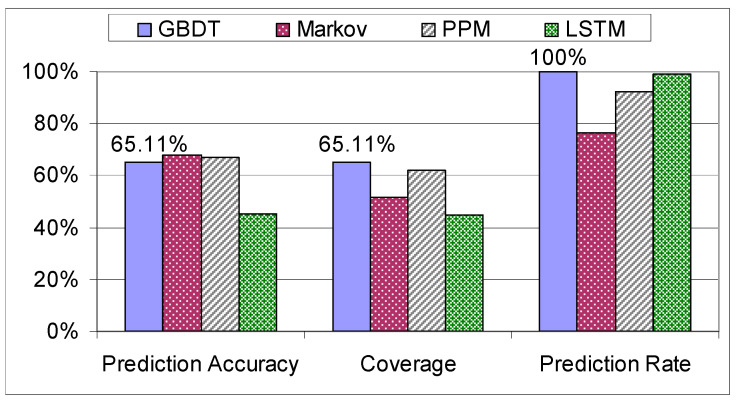
Comparison on the mixed dataset.

In terms of accuracy (calculated based on the number of predictions done), the GBDT outperforms the LSTM but it is inferior to the stochastic approaches taken in [13,14], resulting in an accuracy score of 65.11% (see Figure 16). 

On the other hand, the stochastic methods [14,15] and even the LSTM [16] yield an inferior prediction rate compared to the prediction techniques based on decision trees explored in this paper, as the training data did not contain all the possible assembly sequences, thus making those algorithms unsuitable in scenarios with new data. In contrast, the methods based on decision trees have a prediction rate of 100% since for any input they are able to produce an outcome. The coverage is the main metric that was used when comparing the algorithms as it shows the number of correct predictions out of all the assembly steps from the testing set. We can clearly see that GBDT outperforms the stochastic method with the highest coverage, namely the PPM [15], by 3.24%, while maintaining a prediction rate of 100%. The computational complexities of the analyzed algorithms are not very different: the decision trees have a complexity of $O(f\cdot n^{2}\cdot \log\;n)$, the Markov and PPM models have O(n), whereas the LSTM has a complexity of ${O(n}^{2})$. The method with the highest possible coverage is preferred, because this metric reflects the level of its usefulness in terms of next assembly step suggestions. Consequently, the GBDT is the best suited algorithm for the prediction of the next assembly step.

## 5. Conclusions and Further Work

In this paper, a novel prediction method based on decision trees with ensemble learning was proposed to provide suggestions for the next assembly step. The prediction module is part of a complex sensor-based assembly assistant system whose goal is the guidance of factory workers in the manufacturing process. The evaluation of the proposed method was performed on a dataset collected during the assemblies of 68 trainees, and another one consisting of the assembly processes of 111 experienced factory workers, as well as on their mixture in which the assemblies of the trainees and workers were mixed and randomly sampled. We evaluated the CART, RF, ABDT and GBDT algorithms in terms of prediction accuracy, prediction rate and coverage. The results showed that the GBDT was the best performing among all the evaluated tree-based methods. With a coverage of 65.11% (which is the most important efficiency metric), the GBDT method also outperformed the existing Markovian, PPM and LSTM models. Consequently, the GBDT proved to be a powerful assembly modeling tool, which can be an appropriate prediction component of the assembly assistance system. The utilization of decision tree-based prediction algorithms for assembly process modeling is an original contribution, since the other existing methods rely on stochastic or neural models.

There were several study limitations, mainly, data collection done via physical experiments takes time to set up, namely, preparing the assembly station and restarting the experiment for each participant. The data collection process required several iterations to achieve accurate results that could be used for machine learning purposes.

As future work, a web-based application can be designed to collect more data on the assembly process. The interface would contain the tablet module, the assembly pieces and the same audio and video guidance systems used in the assembly station. Although this would limit the collected data to only assembly sequences, the data can be used to improve the prediction power of the machine learning algorithms. The assembly station interface can also be improved in future studies by using design motivations. In terms of algorithms, we intend to investigate for the next assembly step prediction purpose Dynamic Bayesian Networks, hybrid stochastic predictors, Hidden Markov Models, as well as a method based on the A* algorithm. The predictor with the best assembly modelling capabilities will be integrated into the assembly assistant system.

Finally, to provide an appealing multi-modal training experience tailored for each user through our assembly assistance system, we plan to execute more longitudinal studies considering the predictor, to cover a broad spectrum of user typologies (e.g., age, handedness, disabilities, etc.) and basic emotions or mental states, by conducting experiments involving students from technical and professional high schools (i.e., the future factory operators).

## Data Availability

The data presented in this study are available upon request.
